# Efficacy of a Novel Intracanal Medicament on Total Antioxidant Status in Patients With Apical Periodontitis: A Randomized Controlled Clinical Trial

**DOI:** 10.7759/cureus.54496

**Published:** 2024-02-19

**Authors:** Aishwarya Das, Iffat Nasim

**Affiliations:** 1 Conservative Dentistry and Endodontics, Saveetha Dental College and Hospitals, Saveetha Institute of Medical and Technical Sciences, Saveetha University, Chennai, IND

**Keywords:** apical periodontitis, novel intracanal medicament, total antioxidant capacity, hekla lava, calcium hydroxide

## Abstract

Introduction

Successful endodontic treatment relies upon the microbial debridement of the root canal system. This can be achieved to a great extent by using intracanal medicaments, which inhibit the microbes growing in the root canal. Evaluating the capacity of oxidants in the saliva is a crucial parameter for assessing the antioxidant capacity of any individual, which decreases in inflammatory conditions. An effective intracanal medicament can increase the total antioxidant capacity of saliva, which comes down because of inflammatory conditions.

Aim

To evaluate the effect of two intracanal medicaments on the antioxidant capacity of saliva.

Materials and methods

In a randomized prospective clinical trial, 42 patients with a mean age of 18-70 years were selected based on exclusion and inclusion criteria, and the baseline value of the total antioxidant capacity of saliva was recorded. The patients were categorized into two groups as per the block randomization method (Group I: calcium hydroxide (Maarc, New Delhi, India) intracanal medicament; Group II: Hekla lava (SBL Pvt. Ltd., Germany) intracanal medicament). Access opening, working length determination, and cleaning and shaping were conducted using hand K-files and ProTaper Gold rotary files. Intracanal medicaments were placed according to the groups assigned, and temporary restoration was placed. The patients were recalled after seven days. If the tooth was asymptomatic, obturation was completed, and a saliva sample was collected to assess the total antioxidant capacity.

Results

The total antioxidant capacity of saliva was increased after using intracanal medicaments and endodontic therapy, and there was a statistically significant difference before and after using both the medicaments (p=0.0005; i.e., calcium hydroxide and Hekla lava. When both medicaments were compared, there was no significant difference in the antioxidant capacity of saliva among medicaments (p=0.384).

Conclusion

The total antioxidant capacity of saliva was increased after using both the intracanal medicaments. Hence, Hekla lava can be potentially used as an alternative intracanal medicament.

## Introduction

Apical periodontitis is a bacterial infection of the dental pulp that causes inflammation in the periradicular tissues. The primary goal of endodontic care is to remove microorganisms from the root canal system before root canal filling. However, complete removal of root canal biofilm can be difficult in certain situations. Microbes may spread to apical deltas, isthmuses, root canal ramifications, and dentinal tubules in a long-term infectious phase [1].

During mechanical procedures, some bacteria and bacterial products can penetrate dentinal tubules and into the complexities of the root canal from the smear layer produced [2].

These bacteria might increase in the inter-appointment phase reaching similar pathological levels similar to the beginning of the treatment. To inactivate the inflammatory burden, lasting antibacterial intracanal medicament should be used between appointments until the final sealing of the root canal system [3]. Intracanal medicaments can penetrate areas not reached by irrigants and instruments by staying in the canal for a longer period [4]. Sjogren et al. reported complete radiographic healing in only 68% of root canals that yielded a positive culture before obturation compared with 94% of root canals, which yielded a negative culture [5]. These results concentrate on the importance of completely reducing the bacteria from the root canal system. Various agents were used to extract debris and necrotic pulp tissue, as well as to help kill microorganisms that are not reachable by mechanical instrumentation, during and directly after the root canal treatment [6]. However, calcium hydroxide (Maarc, New Delhi, India) cannot be used as the universal intracanal medication, as it is not effective in the same way for every bacterium that is found in root canals such as Enterococci and yeast and is ineffective for eliminating deeply penetrated bacteria inside the dentinal tubules. Thus, in cases where calcium hydroxide-tolerant microbes are common, an alternative medication may be required to clear the infection and restore healing.

Hekla lava (SBL Pvt. Ltd., Germany) is a fine ash from Mount Hekla that has never been used as an intracanal medicament in vivo. It is available in tablet form commercially. It exhibits a significant zone of inhibition against Enterococcus faecalis and Streptococcus mutans when tried in vitro [7]. The total antioxidant capacity of saliva is a crucial pathogenetic pathway in a variety of endodontic diseases such as apical periodontitis, periapical abscess, and pulpitis [8]. Antioxidant levels in saliva may change as a result of an infection or disease. Saliva can serve as a major diagnostic aid in such pathologies. Reactive oxygen and free radical species are responsible for the host inflammatory response in periodontal pathologies [9]. Hence, comparing the total antioxidant capacity in the saliva of the patients having apical periodontitis using different intracanal medicaments will be useful in proving the efficacy of these medicaments in such cases.

This randomized controlled clinical trial aimed to evaluate the effect of two intracanal medicaments on the antioxidant capacity of saliva. The null hypothesis was that there was no significant difference in the values of the total antioxidant capacity of saliva after using calcium hydroxide and Hekla lava during root canal therapy. The alternate hypothesis is that there is a significant difference in the values of the total antioxidant capacity of saliva after using calcium hydroxide and Hekla lava during root canal therapy.

## Materials and methods

This randomized controlled clinical trial was conducted on patients who reported to the Department of Conservative Dentistry and Endodontics. Institutional ethical board (Institutional Review Board of Saveetha University, India) clearance was already obtained for this study (ref no SRB/SDMDS11/17ODS/08) and was registered with the “Clinical Trials Registry of India” (ref. no. CTRI/2019/05/019420). It was a single-blind study where the patients were not aware of the intervention used. It was structured based on CONSORT guidelines.

For sample size estimation, a pilot study with 10 samples from each group was taken. Based on the results obtained from the pilot study, sample size estimation was calculated using G*Power 3.1.2 software (Heinrich-Heine-Universität Düsseldorf, Düsseldorf, Germany). From the mean and SD obtained from the pilot study, the calculated effect size was 61%, using the repeated measures between factors test, and by keeping the α=5% and power (1-β)=95%, a sample size of 21 for each group was determined, and the total sample size obtained was 42.

Approximately 42 patients in the age group of 18-70 years with single-rooted nonvital teeth having a periapical index score of 1-3 were included in this study. Medically compromised patients, pregnant patients, and patients on antibiotic therapy comprised the exclusion criteria. Randomization was done by the third person not involved in the study using block randomization procedures with unknown block sizes to the investigator until the end of the study. A group was assigned to every serial number based on a random number. For allocation concealment, the sequentially numbered, opaque, and sealed envelope (SNOSE) method was used, which disguised the sequence until interventions were assigned. The dark-colored envelope with the appropriate serial number over it, and a sheet of paper with a randomly generated group number was sealed inside of it. As participants entered the study one at a time, study numbers were allocated to them. The intervention was decided after opening the envelope. The appropriate treatment was administered depending on the group that was designated in the paper.

Initially, the isolation protocol was done with a rubber dam, followed by field disinfection using a swab of 2% glutaraldehyde to prevent contamination. The patient was asked to stay in a resting state for two minutes and asked to spit in a disposable cup. With the help of a syringe, a salivary sample was collected and stored in the Eppendorf tube. The baseline salivary sample (S1) was taken before the commencement of the treatment. Following access cavity preparation, for a collection of microbial samples, a paper point (Dentsply Maillefer, Tulsa, USA) was inserted for 60 seconds and was transferred immediately into an Eppendorf tube (Eppendorf SE, Hamburg, Germany), which contained transport media-thioglycolate broth with vitamin K and hemin. For every sample, four paper points are recorded. Canals were further shaped using K-files (Mani Inc., Utsunomiya, Japan) and ProTaper Gold rotary files (Dentsply Maillefer, Tulsa, USA) with intermittent irrigation using 3% sodium hypochlorite (Prime Dental, New Delhi) and saline (IS Indosurgicals, Coimbatore, India). The canals were shaped until 25.06 taper ProTaper Gold (Dentsply Maillefer, Tulsa, USA) rotary file. Depending on the group determined by opening the sealed envelopes, calcium hydroxide or Hekla lava was used as an intracanal medicament. Calcium hydroxide (Maarc, New Delhi, India) was used in 99% powder form, which was mixed with propylene glycol (Zenvista Meditech, Ghaziabad, India) on a paper pad with an agate spatula until the desired consistency was achieved. Hekla lava (SBL Pvt. Ltd., Germany) was used in the form of tablets, which were triturated in a clean mortar and pestle, and the Hekla lava liquid dilution (SBL Pvt Ltd., Germany) available was mixed with this powdered form to achieve the desired consistency. Medicaments were carried to the canals using a lentulo spiral. The canal was sealed with temporary restoration. The patient was recalled after seven days. If the tooth was asymptomatic, the second salivary sample (S2) was collected from the root canals, and the second microbial sample was collected, following which obturation was completed. If the symptoms persisted, the same intracanal medicament was given for one more week. The salivary samples were stored in a refrigerator and transferred to the laboratory using an icebox. Total antioxidant potential was determined spectrophotometrically using the ferric ion-reducing antioxidant parameter (FRAP) method by measuring total FRAP. This method is based on the reduction of ferric ions in the form of a complex with 2,4,6-Tri(2-pyridyl)-s-triazine (TPTZ) to ferrous ions. The TPTZ-ferrous ion complex has an intense blue color with a maximum absorption at 593 nm wavelength. The color intensity is directly proportional to the concentration of Ferrous ions. To determine the antimicrobial activity of both medicaments, microbiological analysis was done using colony-forming unit count (CFU) and was expressed in CFU/mL.

The data obtained were entered into a spreadsheet and statistically analyzed.

Statistical analysis

The Statistical Product and Service Solutions (SPSS; IBM SPSS Statistics for Windows, Armonk, NY) was used for analyzing the data collected in this study. For describing the data, SD and descriptive statistics mean values were estimated. For finding variations in the bivariate samples for both groups, the t-test sample pairing method was followed, and, for independent groups, the unpaired sample t-test was applied. A probability value of less than 0.05 was used to indicate a significant difference.

## Results

The total CFUs in both groups had decreased after intracanal medicament and endodontic therapy. There was no significant difference between the two groups in terms of reduction of microbial count (intergroup comparison) (Table 1). It was observed that the mean value of the total antioxidant capacity of saliva was increased in both groups after using intracanal medicaments and endodontic therapy (Table 2). In intragroup comparison, there was a significant increase in the total antioxidant capacity of saliva before and after using the medicaments (Tables 3-4). When both the medicaments were compared, there was no significant difference in the antioxidant capacity of saliva among both the medicaments (Table 5).

**Table 1:** Microbial analysis in both groups (CFU/mL) The total CFUs in both groups have decreased after intracanal medicament and endodontic therapy. There was no significant difference between the two groups in terms of reduction of microbial count (intergroup comparison). CFU: colony-forming units

Groups	Mean value of (CFU/mL) of the pretreatment sample	Mean value of CFU/mL of the post-treatment sample after the usage of the medicaments
Hekla lava	1.73 (± 0.76) x10^5^	6.33 (± 1.58) x10^3^
Calcium hydroxide	2.26 (± 0.65) x10^5^	3.87 (± 2.79) x10^3^

**Table 2:** Total antioxidant values of saliva (mg/mL) The above table shows the SD and mean values of the groups. It indicates that the total antioxidant capacity of saliva in both groups has increased after intracanal medicament and endodontic therapy. N: number of samples; SD: standard deviation; SE: standard error

	Groups	No. of samples (N)	Mean value of total antioxidants	SD	SE mean
Before the use of the medicament	Calcium hydroxide	21	0.52	0.17934	0.03913
Before the use of the medicament	Hekla lava	21	0.64	0.49486	0.10799
After the use of the medicament	Calcium hydroxide	21	0.72	0.22511	0.04912
After the use of the medicament	Hekla lava	21	0.82	0.47187	0.10297

**Table 3:** Intragroup comparison of the total antioxidant capacity of saliva with Hekla lava There was a statistically significant difference in the total antioxidant capacity of saliva before and after using Hekla lava intracanal medicament (p<0.05). df: degrees of freedom; SD: standard deviation; SE: standard error; Sig.: statistical significance; t: t-statistic

		SD	Mean	Lower	Upper	SE mean	df	t	Sig. (2-tailed)
Hekla lava	Before and after the use of the medicament	0.22401	-0.1771	-0.27911	-0.07517	0.04888	20	-3.624	0.002

**Table 4:** Intragroup comparison of the total antioxidant capacity of saliva with calcium hydroxide There was a statistically significant difference in the total antioxidant capacity of saliva before and after using calcium hydroxide intracanal medicament (p<0.05). df: degrees of freedom; SD: standard deviation; SE: standard error; Sig.: statistical significance; t: t-statistic

		SD	Mean	Upper	Lower	SE mean	df	t	Sig. (2-tailed)
Calcium hydroxide	Before and after the use of the medicament	0.18951	-0.1938	-0.10754	-0.28007	0.04135	20	-4.686	0.0005

**Table 5:** Intergroup comparison of the total antioxidant capacity of saliva Independent samples t-test was done for multiple pairwise comparisons. It shows that there was no significant difference in the antioxidant capacity of saliva among both the medicaments (p>0.05). df: degrees of freedom; F: F-statistic; SE: standard error; Sig.: statistical significance; t: t-statistic

		Sig.	F	df	t	Mean difference	Sig. (2- tailed)	SE difference	Upper	Lower
Before the use of the medicament	Not assumed equal variances	0.023	5.610	25.164	-1.02	-0.11714	0.317	0.11486	0.11934	-0.35362
After the use of the medicament	Assumed equal variances	0.112	2.634	40	-8.81	-0.10048	0.384	0.11409	0.13010	-0.33106

## Discussion

Highly reactive atoms or molecules known as free radicals can seriously harm the macromolecules. The antioxidant defense system in the human body contains specific mechanisms to guard against the potential effects of free radicals [10]. Oxidative stress is a term used to describe any change in the equilibrium between the defense antioxidant mechanisms and the free radical generation mechanisms. The oxidative stress tends to increase in inflammatory oral conditions including apical periodontitis. To counteract the oxidative stress, the antioxidants prevent further oxidation, hence, the total antioxidant capacity of the body decreases. The levels of these antioxidants normalize once the inflammatory condition comes down [11].

FRAP assay is a quantitative assay for measuring the antioxidant potential within a sample. Ferric iron (Fe3+) is initially reduced by electron-donating antioxidants present within the sample to its ferrous form (Fe2+). The iron-colorimetric probe complex develops a dark blue color product upon reduction which can be measured at 540-600 nm [12]. Hence, the levels of antioxidants can be evaluated before and after treatment using this assay.

Intracanal medicaments are used in apical periodontitis to lower the infection in the canals; consequently, the total antioxidant capacity of saliva also increases later after the inflammation comes down [13]. Calcium hydroxide stimulates tissue mineralization. The alkaline phosphatase function is activated by higher pH levels, which aids in the formation of hard tissue. The presence of hydroxyl ions creates an alkaline state that aids in early recovery and also possesses antimicrobial properties [14].

The total antioxidative activity of saliva was lower among patients with chronic periodontitis before root planning compared to the control group [15]. Oxidative stress was increased in patients after composite restorations because of sustained monomer release from composite restorations [16]. The antioxidant activities of the saliva were reduced with an increase in the level of inflammation [17].

Hekla lava is a fine ash obtained from Mount Hekla, situated in Iceland. It can be used to treat many oral problems, such as gingivitis, abscess of the gums, toothache, and so on. Moreover, it can be used as a dressing for the management of the dry socket of the third molars [18]. It was used for the first time as an intracanal medicament in the present study. In an in vitro study, it has shown a significant zone of inhibition against strains of Enterococcus faecalis and Streptococcus mutans [18].

No clinical trials have been reported wherein Hekla lava was used as an intracanal medicament. From this research, it was noted that Hekla lava had a significant effect on the total oxidant status of saliva before and after using this medicament. This may be attributed to the presence of large amounts of sulfur, silica, lime, magnesia, ferrous oxide, and fluoride. It has an anti-inflammatory effect that helps in treating gingivitis, chronic periodontitis, and so on [18].

The intracanal medicaments are used in apical periodontitis to lower the infection in the canals. It was observed that after biomechanical preparation of root canals, 40% of the root canals still contained various species of bacteria [5]. Ideally, the intracanal medicament should remain inside the root canal for at least seven days to eliminate the microbes that can survive even after biomechanical preparation [19]. We evaluated the antimicrobial activity of both the medicaments by assessing the total CFU/mL. It was observed that, in both groups, the total microbial load was decreased after intracanal medicament and endodontic therapy.

Apical periodontitis-related bacteria and their metabolites trigger the local immune system, which may lead to low-grade chronic inflammation and eventual localized periapical bone deterioration. This could have an impact on systemic health [20]. The serum levels of inflammatory markers were reported to be raised in apical periodontitis and were reduced after endodontic treatment [21]. It was found that some biomarkers can be measured in saliva, namely, CRP and FGF-23, and may serve as potential prognostic biomarkers [22].

This is the first study where the total status of the antioxidant levels of saliva was checked after endodontic therapy. It was observed that after root canal therapy and usage of intracanal medicaments in symptomatic apical periodontitis cases, total antioxidant capacity was increased irrespective of the medicaments used. Hence, the null hypothesis was rejected, and an alternate hypothesis was accepted as there was a significant difference in the antioxidant capacity of saliva before and after using the medicaments. However, there was no significant difference between the groups.

The limitations of the present clinical trial include the trial having been conducted in a particular area with a small group of people and the lack of a negative control. Future research should focus on studies with larger sample sizes, and studies analyzing the correlation of salivary with serum biomarkers should be performed.

## Conclusions

According to the results of the present clinical trial, Hekla lava is quite effective in increasing the total antioxidant capacity of saliva and is comparable to the gold standard calcium hydroxide. Hence, it can be used alternatively as an intracanal medicament in symptomatic apical periodontitis cases. Further clinical trials should be conducted to validate the findings of the present study.
